# Regulation and dysregulation of microRNA - transcription factor axes in differentiation and neuroblastoma

**DOI:** 10.1007/s00018-025-05832-4

**Published:** 2025-08-08

**Authors:** Fakhira H. Nazki, Cameron P. Bracken

**Affiliations:** 1 Laboratory Head - Gene Regulatory Networks Laboratory, Centre for Cancer Biology, University of South Australia, Bradley Building, Rm HB-9-31, North Terrace, Adelaide, South Australia 5000 Australia; 2 School of Biological Sciences, Faculty of Sciences, University of Adelaide, Adelaide, SA Australia

**Keywords:** Neuroblastoma, MicroRNA, Transcription factor, Cancer, Gene regulation

## Abstract

Development is characterized by dynamic changes in gene expression as cells traverse genetic pathways and make lineage-specific commitments. Transcription factors, which drive gene expression, and microRNAs, the largest class of post-transcriptional regulators, often function together within the same genetic networks. These interactions frequently include direct regulation of one another and shared target genes, forming feedback and feedforward loops that fine-tune gene expression to establish and maintain cell identity. The interplay between transcriptional and post-transcriptional regulation is particularly extensive during development, where disruptions in gene expression programs can cause cells to become trapped in immature proliferative states that result in paediatric cancers. This review focuses on the intricate cross-regulation between transcription factors and microRNAs, highlighting their contributions to developmental cancers with a particular emphasis on neuroblastoma, the most prevalent extracranial solid tumour in children, which arises from the failure of neural crest-derived cells to properly differentiate during sympathoadrenal development.

## Introduction to neuroblastoma

At the beginning of the 20th century, cancer was recognized as a genetic disease, characterized by chromosomal alterations that lead to uncontrolled cell proliferation. Over time, this understanding has evolved to also view malignancy as a consequence of incomplete differentiation. According to this perspective, cancer cells fail to follow their normal developmental path towards stable, specialized states, instead becoming “trapped” in immature, proliferative states that are then subject to natural selection and clonal expansion which drives tumour growth and progression [1, 2]. The connection between differentiation and cancer is especially prevalent in paediatric cancers such as Neuroblastoma (NB), the most common extracranial solid tumour in children. Predominantly diagnosed around 18 months of age [3], NB constitutes 6–10% of childhood cancers but is responsible for 12–15% of all paediatric cancer deaths [3–5]. In contrast to adult cancers linked to aging [6–8], NB emerges as a developmental disorder originating from neural crest cells (NCCs) - a multipotent, stem-like population that ultimately gives rise to a diverse array of tissues at distal sites [9, 10]. Beginning with delamination from the neural tube, NCCs undergo epithelial-mesenchymal transition (EMT), which promotes a migratory state. Cells then travel to distant regions throughout the body, guided by specific molecular cues, and differentiate along various lineages to form tissues including craniofacial cartilage, smooth muscle, melanocytes, enteric neurons, and the ganglia of the peripheral nervous system [11, 12]. Throughout this migration and differentiation, NCCs navigate specific trajectories and traverse developmental bottlenecks before committing to distinct cell fates, a process tightly regulated by networks of gene regulators [13–16].

NB exhibits considerable heterogeneity with regard to clinical outcomes, ranging from tumours that regress spontaneously to aggressive forms resistant to multiple treatment modalities [17]. These different outcomes are likely explained, at least in part, by extensive heterogeneity in the nature of the underlying genetic alterations that can occur to an array of transcription factors (TFs), chromatin remodellers, and kinases. However, the presence of these genetic alterations alone does not generally predict disease trajectory or adequately inform treatment strategies. An exception is MYCN amplification which may suggest more aggressive treatment choices as it is consistently associated with poor prognosis [18], however, it is only a characteristic of ~ 25% of all NB cases [19]. Other common alterations, like TERT rearrangements (present in ~ 25% of cases) or ALK amplification (< 10% of cases), show a broad range of clinical outcomes, limiting their reliability as standalone predictors of disease severity [20, 21].

Interestingly, when NB is diagnosed in infants under 18 months of age, and especially when those tumours are more differentiated or classified as stage 4S (a category defined by limited metastatic spread), spontaneous regression often occurs. This favourable outcome is linked to genomic profiles marked by whole chromosome gains rather than segmental aberrations, contributing to a less aggressive tumour biology [22]. This may be surprisingly prevalent, as autopsies of infants who had died from cancer-independent causes, suggest the rate of spontaneous, undiagnosed regression is far higher than the number of cases that are identified which then resolve [23–25]. Given the potential severity of a NB diagnosis, one may argue for erring on the side of aggressive treatment, however, aggressive treatments in children can cause debilitating life-long consequences, including hearing loss, endocrine abnormalities, growth impairments, organ toxicity and increased risk of developing secondary malignancies [26–28].

Although, NB was first characterised in the 19th century (for an excellent historical perspective, see [29]), our understanding of its molecular underpinnings has only been elucidated in recent years with the advent of bulk, then single-cell sequencing. Analysis of normal, developing human adrenal glands (the most common site of the origin of NB) has revealed a developmental trajectory in which intra-adrenal sympathoblasts and chromaffin cells are derived from nerve-associated schwann cell-like precursors that passage through intermediate bridging cell states [9, 30]. Single cell sequencing has also informed the nature of infiltrating immune cells and the existence of mesenchymal-like NB cells whose presence is difficult to disentangle from bulk-seq data, but which can now be discerned at the single cell level. As we will discuss later, NB can be classified along an adrenergic (ADRN) to mesenchymal (MES) axis which includes an aggressive intermediate cell state [31, 32]. Such plasticity is conserved in bone marrow metastases [33], and tumours with a mesenchymal, less differentiated signature represent a more aggressive, though rarer subtype [31, 34–36]. For instance, a standardised transcriptomic atlas of NB tumours, integrating seven single-cell datasets, revealed high heterogeneity in cell cycle genes, a small MES subtype proportion, and markers of advanced differentiation in lower-risk patients [36]. Dozens of separate immune cell subtypes have also been identified infiltrating NB [37], which tend to be characterised by immunosuppressive macrophages, dysfunctional natural killer (NK) cells and a largely normal, though low abundance, T cell infiltrate whose dysfunction increases after chemotherapy [37–39].

Even though single-cell sequencing is revolutionising our understanding of developmental trajectories and providing a refined understanding of differentially expressed genes in various cell types and sub-populations, the importance of specific genetic drivers was identified more than a decade before these technological advances. TFs directly govern gene expression and thus, it is not surprising that many prominent drivers of NB, such as MYCN and PHOX2B [40–42], are TFs. Indeed, many TFs are capable of regulating pluripotency and differentiation more generally. For example, a screen that systematically over-expressed thousands of TF isoforms and mapped effects on cell fate via single cell transcriptomics revealed about a quarter of all TFs are capable of either inhibiting or driving embryonic stem cell differentiation [43]. However, transcription is not the sole determinant of gene expression as there are multiple layers of regulatory control beyond TF binding and activity. Prominent among these regulatory factors are microRNAs (miRNAs), sequence-specific repressors of gene expression that act as the target recognition component of the RNA-induced silencing complex (RISC). As the largest family of post-transcriptional gene regulators, miRNAs have a dynamic relationship with TFs, many of which are themselves direct miRNA targets [44]. Additionally, TFs often regulate the transcription of miRNAs that, in many cases, modulate their own expression, establishing feedback loops that can enhance, suppress, or buffer gene expression programs during development and differentiation [45, 46]. Through such mechanisms, miRNAs play a fundamental role in guiding developmental transitions, ensuring that cellular processes unfold with both spatial and temporal precision [47].

This review aims to explore the intricate interactions between miRNAs and TFs within signalling networks, elucidating how these interactions regulate gene expression at both post-transcriptional and transcriptional levels. In so doing, we focus on NB, a developmental cancer where differentiation goes awry as cells transverse through various genetic bottlenecks and cell fate decision points - the very situations that are often subject to miRNA/TF feedback regulation.

### MicroRNAs

miRNAs are short non-coding RNA molecules, around 22 nucleotides in length. They regulate gene expression by binding complementary sequences in target RNAs, typically in the 3’ untranslated region (3’UTR) of messenger RNAs (mRNAs). This binding has a suppressive effect on gene expression by either promoting degradation of the mRNA or inhibiting its translation. The synthesis and mechanism of action of miRNAs has been extensively described in previous reviews [48] and will only be briefly discussed here.

MiRNA biogenesis begins in the nucleus with the transcription of primary miRNAs (pri-miRNAs) by RNA Polymerase II or III. The region of the pri-miRNA that will ultimately give rise to the mature miRNA forms a hairpin structure on account of intra-strand base pairing. The hairpin is then recognised and processed by the Drosha-DGCR8 complex which cuts both strands of the locally paired RNA structure at its base, forming precursor miRNAs (pre-miRNAs) which are exported to the cytoplasm by Exportin 5 and Ran-GTP [49]. In the cytoplasm, the RNase enzyme DICER cleaves the loop, forming miRNA duplexes [50] of which one strand (often called the “guide”) is loaded into one of four Argonaute (AGO)-family proteins (in mammals) to form the RISC, along with additional accessory proteins [51]. The mature miRNA then serves as the target-recognition component of RISC, guiding it to specific mRNAs via complementary base pairing, most often within the 3’ UTR. Recent studies reveal that miRNAs can also regulate gene expression by targeting the coding sequence [52], however for such interactions to result in effective gene suppression, unusually extensive sequence complementarity between the miRNA and its target is required [53].

### MiRNAs: regulators of both individual targets and collective regulators of biological processes

A central feature of miRNA is the surprisingly short (~ 6–7 nucleotide) “seed” sequences through which they identify their targets. The minimal nature of this target interaction interface, compounded by binding- dependent AGO2 phosphorylation [54], means most interactions are unstable, and only result in modest repression. This however can be vastly increased artificially through exogenous miRNA over-expression [55]. Endogenously, for stronger repression to be exerted, extended seed pairing (across ~ 8 nucleotides) may be required, as may utilising additional interaction interfaces toward the 3’ end of a miRNA [56] which are made accessible by conformational changes that occur after the initial seed binding [57]. Therefore, for a miRNA to exert functional effects, an obvious strategy is to target a gene through a longer interaction interface and thus create a more stable binding event. However, a second possibility for functionally significant effects is created by the short nature of the interaction interface, meaning a single miRNA has thousands of potential targets. Thus, to exert biologically meaningful interactions, a single miRNA, or multiple miRNAs from the same co-regulated miRNA cluster may simultaneously regulate multiple genes. This may be especially important if the genes themselves encode proteins that are components of the same complex or signalling pathway, as the sum total of even modest interactions may add up to biologically meaningful effects [55, 58].

**Fig. 1:**
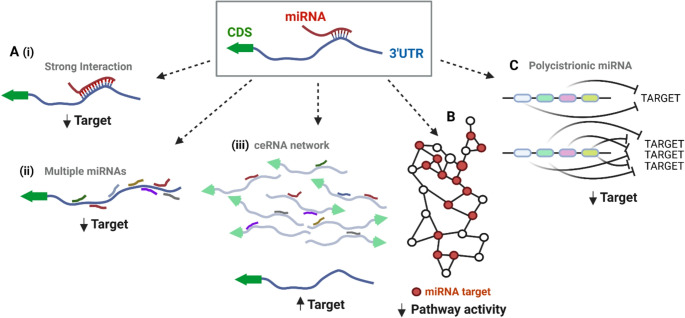
Key factors affecting microRNA function. The capacity for miRNAs to regulate target transcripts and processes is dependent upon (**A**) (i) stability of the interaction interface which is increased by base pairing extending beyond the seed (nt 2–8), (ii) co-targeting by multiple miRNAs and (iii) availability of the miRNA which is influenced by the abundance of competing transcripts. The functional significance of miRNA targeting can also be enhanced by (**B**) multi-node targeting within a biological pathway and (**C**) co-expression of multiple miRNAs with shared targets through the organisation of miRNAs in one polycistronic transcript

Figure 1 illustrates these concepts. In Fig. 1A, simplified one-on-one miRNA-target interactions are contingent upon factors such as miRNA and target mRNA abundance, binding strength and the presence of competing endogenous RNAs (ceRNAs) that can sequester miRNAs and thereby modulate their availability and efficacy. In Fig. 1B, all target interactions are still contingent upon the same factors, but meaningful regulation of a biological process may be enhanced if the target genes themselves are interconnected [59–62]. Co-targeting by multiple miRNAs have also created an evolutionary incentive to locate miRNAs within polycistronic clusters which come under a single regulatory control (Fig. 1C). The miR-200 family for example, consist of five miRNAs located over two chromosomal loci that collectively, target two separate classes of gene based upon a single nucleotide difference within their seed region [63]. These miRNAs play especially important roles in development as the mutually repressive feedback loop in which they participate with the ZEB TFs form a key regulatory axis in EMT [64–67], a pre-requisite to enable cell motility in such early processes as gastrulation and neural crest delamination (which begins the process of sympathoadrenal differentiation from which NB typically arises).

Perhaps the best-studied example of polycistronic miRNAs however is the miR-17 ~ 92 cluster, often referred to as oncomiR-1 due to its frequent association with cancer. This cluster is essential for normal development and its dysregulation results in severe developmental defects, whilst its complete absence is lethal postnatally [68]. Collectively, there are 15 separate miRNAs within the miR-17 ~ 92 family, spread across 3 chromosomal locations, and grouped into 4 separate target classes on the basis of shared seed-sequences. One mechanism through which oncogenic effects are exerted is via coordinated regulation of the PI3K/AKT signalling pathway, with each member contributing modestly to the repression of specific targets, but achieving a strong biological impact on growth, survival and metabolism through their collective action. Specifically, miR-19a and miR-19b suppress the expression of PTEN, a tumour suppressor that counteracts PI3K activity. Reduced PTEN levels lead to an increase in PI3K-mediated signalling, which in turn promotes AKT activation. Simultaneously, miR-17 and miR-20a target regulatory subunits of Protein Phosphatase 2 A (PP2A), a phosphatase that dephosphorylates and inactivates AKT. By suppressing PP2A, these miRNAs ensure sustained phosphorylation and activation of AKT [69].

As with many cancers, miR-17 ~ 92 overexpression is frequently observed in NB, either through gene amplification or MYCN-mediated transactivation [70, 71], where it is linked to poor prognosis [72]. Its promotion of NB is likely attributed to the modulation of multiple signalling pathways including AKT (described above), the Wnt pathway through direct repression of the Wnt modulator DKK3 [73, 74], and the suppression of TGF-β signalling via multiple miR-17-92 family members targeting multiple TGF-β pathway components [75]. Accordingly, the inhibition of miR-17-5p was reported to abolish the growth of MYCN amplified and therapy resistant NB cells in an orthotopic tumour model [76].

The clustering and co-regulation of multiple miRNAs thereby underscores a critical aspect of miRNA function: their ability to regulate development and disease through the collective, and sometimes individually mild, regulation of multiple functionally related genes. In this way, they can coordinate multiple cellular pathways to maintain cellular state and function, respond to physiological changes, or guide differentiation decisions.

### Challenges in identifying critical MiRNA interactions

Because miRNAs have the potential to modulate thousands of genes, a significant challenge is presented in discerning which are biologically significant, especially with the development of CLIP (cross-linked immunoprecipitation)-seq and related assays which make it possible to identify miRNA – mRNA interactions en masse [77, 78]. Additionally, the issue of stoichiometry, the relative abundance of miRNAs and their target mRNAs, also plays a crucial role. If the target mRNA is present in significantly larger amounts than the miRNA, the regulatory impact of the miRNA might be minimal [55]. This might not be obviously apparent when analysing AGO pulldown data as inefficiency in cross-linking and base-pairing specificities between different miRNAs mean results are not quantitative. Thus, what is the significance of a miRNA: target interaction detected a handful of times among millions of sequencing reads, especially if the target itself is highly expressed?

Another challenge lies in interpreting the significance of genuine miRNA-target interactions when these interactions primarily serve a “buffering” role rather than acting as prominent molecular switches. Random fluctuations in gene expression due to internal or external factors add ‘noise’ or variability, which miRNAs play a crucial role in stabilizing [46]. Using both single-cell reporter assays and mathematical modelling, it was found that for lowly expressed genes, and in circumstances where multiple miRNAs target the same transcript, protein expression noise is buffered by the cumulative actions of miRNAs [79]. Paradoxically however, variation may increase for highly expressed genes. This property may have direct relevance to differentiation as even in the absence of external stimuli, stem cells exist in multiple states which enables their diversification into different cell types. These different states exist in part due to the differential expression of cell-to-cell variable miRNAs (such as miR-182) which have the propensity to target pluripotency TFs (such as NANOG) and genes co-expressed with NANOG via a plethora of mostly weak interactions. Cell specific differences in miRNA expression increase cell-to-cell variation in pluripotency related targets, ultimately promoting future diversification into new cell states which is delayed by the loss of miRNA activity [80]. Indeed, there are extensive links between miRNAs and the Yamanaka factors (OCT4, SOX2, KLF4, MYC) which are capable of re-programming cells into a pluripotent state, but which do so less efficiently in stem cells lacking DGCR8, a protein required for canonical miRNA biogenesis [81]. The Yamanaka TFs also occupy the promoters of most of the miRNA genes that are preferentially or uniquely expressed in embryonic stem cells [82], further supporting a role for miRNAs and their interconnected relationship with TFs at the heart of stem cell maintenance and differentiation.

### MicroRNA – transcription factor regulatory networks

The ultimate results of miRNA-mediated gene regulation are complex, representing the sum total of a multitude of weak and strong effects as miRNAs, working both individually and co-operatively, target the majority of protein-coding genes. Among these, however, TFs represent an especially important type of miRNA target due to the major role they play in orchestrating gene expression across biological systems. Since each TF regulates the expression of many downstream genes, miRNA-mediated control of TFs can trigger cascading effects on cellular pathways and processes. Supporting this, both Gosline et al. (2016) and Pillman et al. (2019) found that changes in gene expression induced by the perturbation of miRNAs occur predominantly at the level of transcription, rather than post transcription where the direct effects of miRNAs are exerted. In other words, although the direct effect of a miRNA is post-transcriptional, much of their influence on gene expression is mediated indirectly at the level of TF activity [44, 83].

TFs may be especially primed to be regulatory targets of miRNAs as transcripts encoding TFs are typically present at lower levels than is the case with other gene types [84], meaning stoichiometric ratios may be favourable to miRNA-mediated regulation as miRNAs are comparatively abundant. Further, regulation is two-way, with miRNAs both post-transcriptionally regulating TFs, and themselves being subject to their own transcriptional regulation, often by the same TF [85, 86]. In fact, TFs which are themselves highly connected in genetic networks, tend to regulate more miRNAs than one would expect by chance, and are themselves more likely to be regulated by multiple miRNAs. Such interactions form different types of “mini networks” (known as gene regulatory network motifs, GRNs) which buffer, fine-tune, switch, or lock-in gene expression programs depending upon the nature of the specific co-regulation. Such reciprocal regulation may be especially suited to a differentiation context in which cells transition between states or move in more than one direction, depending on the activities of key TFs that set their transcriptional programs.

#### Reciprocal feedback loops (RFLs)

**Fig. 2:**
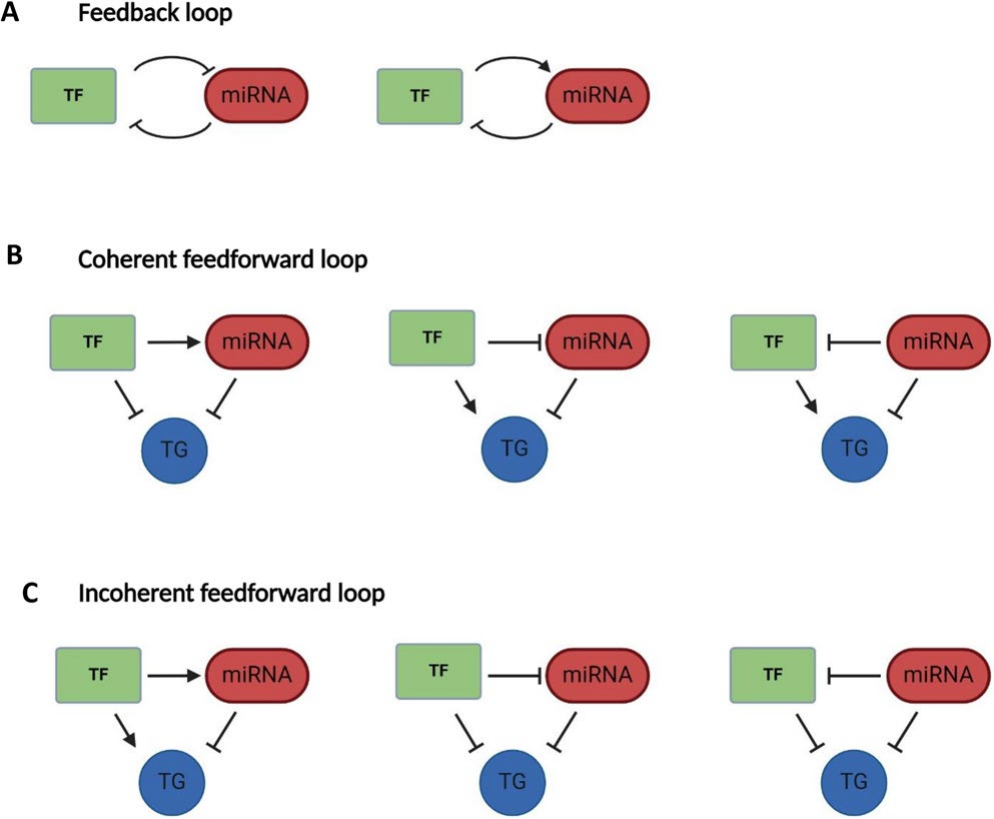
Gene regulatory motifs involving both microRNAs and transcription factors. (**A**) MiRNAs and TFs are frequently co-associated in feedback loops in which the miRNA directly inhibits the TF and the TF, either positively or negatively, affects transcription of the miRNA. When co-regulation of a shared target gene is involved, the motif can be classified as either (**B**) coherent, whereby the miRNA and TF have the same outcome on gene expression or (**C**) incoherent, where the actions of the miRNA and TF oppose each other

In RFLs, two genes are locked in mutual regulation, which is often used to establish bidirectional control and toggle between possible cell states (Fig. 2A) [87]. In many cases, feedback loops involve a miRNA and a TF and where the nature of the feedback is mutually repressive, alternate cell states can be driven. Mutually repressive feedback between miR-34a and MYCN represents a NB-relevant example [88, 89], as does feedback between the miR-200 family and the ZEB TFs [64–67] which govern the EMT phenotypic switch that precedes cell motility in a variety of developmental contexts, including NCC migration.

It is also possible that the participant TF may activate transcription of a miRNA that is directly repressing it, and thus promote homeostasis by effectively limiting its own expression after it increases past a certain threshold. Of relevance to neurogenesis, in midbrain dopamine neurons the TF Pitx3 activates a battery of genes critical for dopamine neuron maturation, whilst simultaneously inducing the transcription of miR-133b, which post-transcriptionally represses Pitx3 [90]. Similarly, midbrain development and the size of the dopaminergic progenitor pool is controlled by feedback between miR-135a2 and the TF Lmx1b. Lmx1b activates miR-135 and is itself a target of miR-135 repression. The impact of this regulation then extends further to the targeting of multiple genes within the Wnt pathway which are themselves activated by Lmx1b and repressed by miR-135 [91]. In so doing, the GRN is extended to include targeting of shared genes, forming feedforward loops (FFL).

#### Feedforward loops (FFLs)

**Fig. 3:**
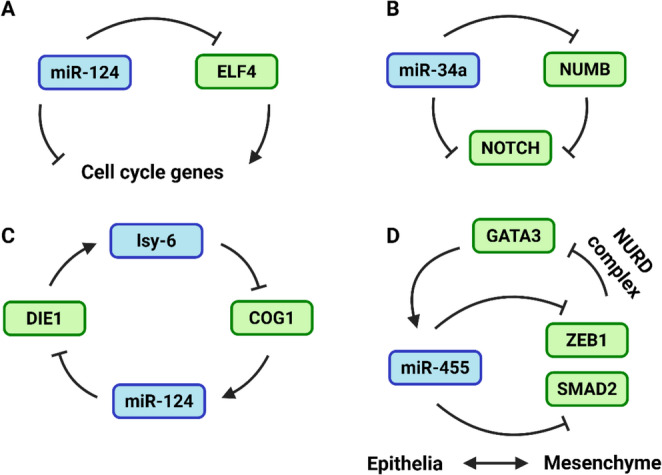
Examples off feedback and feedforward interactions. Examples of (**A**) coherent and (**B**) incoherent feedback between a miRNA and TF and **C**, **D**) examples of complex feedback regulation involving multiple miRNAs and/or TFs that are active in developmental or differentiation processes

FFLs involve three components: a TF, a miRNA, and a target gene (TG). In this motif, the TF regulates both the miRNA and the TG, while the miRNA also directly regulates the target gene. FFLs can be either coherent (Fig. 2B), or incoherent (Fig. 2C), depending upon the outcome of regulation. With coherent FFLs, the miRNA and TF reinforce each other’s activities which ensures robust and decisive gene expression [92–94]. For example, miR-124 promotes neurogenesis by both directly targeting cell cycle genes and by repressing ELF4, which otherwise promotes an undifferentiated state through the upregulation of many of these same cell cycle targets (Fig. 3A) [95]. Similarly, the aforementioned activation of miR-17 ~ 92 transcription by MYCN constitutes a coherent FFL, where members of the miR-17 ~ 92 cluster target genes involved in cell cycle inhibition and apoptosis, thus reinforcing the proliferative and survival signals driven by MYCN [76]. In contrast, incoherent FFLs (IFFL) see the miRNA and TF exerting opposing effects on the target gene. One such example is the mutual repression of NOTCH by miR-34a and NUMB (a miR-34a target), buffering gene expression noise and creating a robust binary switch for Notch expression, critical during symmetric/asymmetric division of cancer stem cells (Fig. 3B) [96].

FFLs can also take on additional complexity, involving multiple TFs or other genes to achieve the same regulatory ends. For instance, in *Caenorhabditis elegans*, neuronal fate decisions in taste sensor neurons involve a double negative feedback loop between miRNAs (lsy-6, miR-273) and TFs (Die1, Cog1), promoting bistability from a common precursor through unstable hybrid states (Fig. 3C) [97]. Another example is miR-455-3p which inhibits EMT and TGF-β signalling through direct targeting of ZEB1 and SMAD2, in turn exacerbated by ZEB’s inability to recruit the repressive NURD complex to the GATA-3 promoter. As GATA-3 transcriptionally activates miR-455-3p gene expression, the signal is thereby propagated (Fig. 3D) [98].

Although not strictly an investigation into FFLs, it is of note that for about half of all miRNAs examined, miRNA targets were found to be significantly enriched among the transcriptional targets of at least one TF [99], underscoring widespread TF-miRNA interactions in gene regulatory networks (GRNs). Given the numerous TFs, miRNAs, and genes in sympathetic neurogenesis and NB, hundreds of regulatory FFLs and related loops (RFLs) likely operate across these developmental cancers.

### MicroRNAs, transcription factors and neuroblastoma

Gene regulatory networks are complex; however, an argument can be made that in a multipotent system such as NCCs, regulatory complexity may be especially essential because cancer is a consequence of the failure to exert proper regulation as cells navigate various developmental paths, bottlenecks and decision points that are inherent in developmental systems. Multiple TFs are required throughout these different stages, which in turn require networks of fine-tuners and amplitude modulators acting at the post-transcriptional (miRNA) level. From such complexity, precise spatiotemporal control of gene expression is made possible to ensure correct cell differentiation [100, 101].

With specific regard to NB, the contribution of miRNAs has been extensively studied. For example, a PubMed search involving the keywords “miRNA” and “NB” returns over 800 results. These include the global profiling of microRNAs across NB cell lines and patients [72, 102–108], and bioinformatic efforts to overlay miRNA target prediction with anti-correlated mRNA and miRNA expression to identify miRNA-centric regulatory hubs [109–114]. However, as with all aspects of miRNA research, discerning impactful examples of regulation from the RNA milieu is challenging and studies that are reliant upon miRNA over-expression are probably best viewed as indicating what a miRNA is potentially capable of doing, rather than taking at face value experiments involving exogenous expression as being truly indicative of endogenous function. This is because one can “force” effects (weak targets become strong) when miRNAs are expressed to levels beyond which they reach endogenously. Table 1 provides a curated selection of miRNAs in NB, focusing on studies with robust experimental evidence. Only miRNAs supported by knockdown and rescue experiments, target validation (e.g., luciferase reporter assays, CLIP-seq, qPCR, Western blotting or RNA sequencing), and detailed functional characterization (e.g., effects on proliferation, differentiation, or chemoresistance) are included.

**Table 1:** Experimentally-supported MiRNA targeting in neuroblastoma

miRNA	Target (s)	Experimental validation	Key Findings	Reference
miR-1304-5p	NRAS, RRAS, PTPN11, IQGAP1	- CRISPR-Cas9 screen - miRNA inhibitors/mimics - Rescue experiments - Luciferase reporter assays	- Enhances ALK TKI sensitivity, induces apoptosis via RAS/MAPK pathway - Therapeutic synergy with ALK inhibitors in vivo.	[115]
miR-99b-5p, miR-380-3p, miR-485-3p	PHOX2B, LIN28B, FGFR3, NOTCH2, EMT-related genes	- High-throughput functional screen for chemo sensitization - RNA sequencing for target identification and pathway analysis - siRNA knockdown and rescue experiments for direct miRNA targets - Luciferase assay for direct PHOX2B targeting - Validation in multiple NB models, including patient-derived xenografts (PDXs)	- miRNAs sensitize NB cells to doxorubicin, enhance apoptosis, and reduce proliferation by targeting key oncogenes and chemoresistance pathways. - miR-99b-5p directly represses PHOX2B and LIN28B, while miR-380-3p and miR-485-3p regulate FGFR3, NOTCH2, and EMT pathways, collectively reprogramming cells toward a less chemoresistant phenotype.	[104]
miR-449a, miR-2110, miR-137, miR-124-3p, miR-34a-5p	MYCN (via direct and indirect mechanisms)	-PCR and Western blot for MYCN expression - Luciferase reporter assays (for miR-449a, miR-34a-5p) - siRNA knockdown and overexpression - Differentiation markers (e.g., βIII-tubulin, NSE, GAP43) - Neurite outgrowth assays - Clinical dataset correlation analysis between MYCN and miRNAs	- miR-449a and miR-34a-5p directly target MYCN 3′UTR, promoting differentiation - miR-506-3p, miR-124-3p, and miR-2110 inhibit MYCN through indirect pathways - miR-2110 and miR-137 expression are anti-correlated with MYCN levels in clinical NB specimens - Combined MYCN knockdown and miRNA overexpression enhance differentiation and reduce NB cell viability.	[116]
miR-15a, miR-15b, miR-16	MYCN	- qRT-PCR and Western blot for MYCN mRNA and N-Myc protein - Luciferase reporter assays - AGO2 RNA-IP - Xenograft models - MTT, migration, colony formation assays	- Direct MYCN targeting - miRNA overexpression suppresses proliferation, migration, invasion, and tumour growth in xenografts - Downregulation correlates with higher MYCN levels and poor prognosis	[117]
miR-204	MYCN	-MYCN knockdown in multiple NB cell lines. - Doxycycline-inducible miR-204 expression system in MYCN-amplified cells. - Chromatin immunoprecipitation (ChIP) confirming MYCN binding to the miR-204 promoter. - Biotin-miR pulldown to show direct binding of miR-204 to MYCN mRNA. -In vivo tumour suppression	-MYCN binds and represses the miR-204 promoter - miR-204 directly targets MYCN mRNA - miR-204 reduces NB cell proliferation and colony formation in vitro and delays tumour growth in vivo. - miR-204 acts as a tumor suppressor by establishing a double-negative feedback loop with MYCN in NB cells.	[113]
miR-15a/16 − 1	BCL2, CCND1	- miRNA knockdown (antagomiRs) and expression (mimics) - BL-8040 treatment to inhibit CXCR4. - In vivo xenografts	- BL-8040 increases miR-15a/16 − 1 levels, reducing BCL2 and CCND1 expression. - CXCR4 inhibition impairs NB cell survival and proliferation. - miR-15a/16 − 1 is crucial for CXCR4-mediated therapeutic effects.	[118]
miR-424-5p, miR-503-5p	ALK	- Genome-wide miRNA profiling - Luciferase assays for direct ALK targeting (miR-424-5p) - miRNA mimics - Western blot for ALK -protein expression	- miR-424-5p directly targets ALK, reducing ALK protein in NB cells. - miR-503-5p indirectly downregulates ALK - miR-424-5p and miR-503-5p reduce NB cell viability.	[119]
miR-506-3p	PLAGL2 (indirectly regulates MYCN)	- miRNA antagomirs and mimics - Luciferase reporter assays (PLAGL2 is a direct target of miR-506-3p) - CHIP-PCR (PLAGL2 binds MYCN promoter) - Western blot - Cell viability, differentiation, neurite outgrowth assays	- miR-506-3p directly targets PLAGL2 - PLAGL2 directly promotes MYCN transcription - Knockdown of PLAGL2 induces NB cell differentiation and reduces proliferation. - Retinoic acid increases miR-506-3p, represses PLAGL2 and MYCN, and promotes differentiation.	[120]
miR-182-5p, miR-203a, miR-222-3p, miR-432-5p	Multiple miRNAs regulated indirectly by p53	- p53 Knockdown and activation (nutlin-3) - miRNA mimics -Growth, apoptosis (PARP cleavage), differentiation assays - qPCR measuring miRNA expression.	- Four miRNAs upregulated after p53 activation (via nutlin-3 treatment). - miR-182-5p had the strongest anti-proliferative effect, induced apoptosis, and promoted neuronal differentiation. - All miRNAs reduced proliferation in p53 wild-type NB cells.	[121]
miR-184, let-7a, miR-92, miR-107, miR-181b	Multiple mRNAs	- MYCN knockdown (siRNA) - PCR (miRNA expression). - ATRA Treatment - miRNA mimics - Monitoring cell cycle and apoptosis using FACS and Caspase assays.	- miR-184 expression induces apoptosis and G1 arrest, with a stronger effect in MYCN-amplified cells. - miRNA profiles correlate with NB subtypes, prognosis, and MYCN regulation. - Similar effects on miRNA expression with ATRA and MYCN knockdown.	[102]

The following sections delve into the interactions between key TFs and miRNAs in NB, highlighting how these relationships influence the disease’s pathogenesis and progression. By concentrating on key TFs, especially MYCN and PHOX2B, we aim to demonstrate the complex regulatory landscape that underpins differentiation and highlight how developmental cancers can arise when these regulatory circuits break.

### MYCN - miRNA interaction

MYCN is a proto-oncogene that belongs to the MYC family of TFs which also includes MYC and MYCL. While MYC alterations are observed in a broad array of cancers, MYCN is especially implicated as a key factor in paediatric cancers originating from central and peripheral nervous system tissues [122]. In fact, MYCN amplification, which can reach levels of more than 10 copies per genome, is a hallmark of high-risk NB (occurring in ~ 22% of cases) [4, 123]. The MYCN gene encodes N-myc, which is critical in the regulation of growth, metabolism, and cellular differentiation. The consequence of its overexpression is proliferation and the maintenance of undifferentiated states [124].

**Fig. 4:**
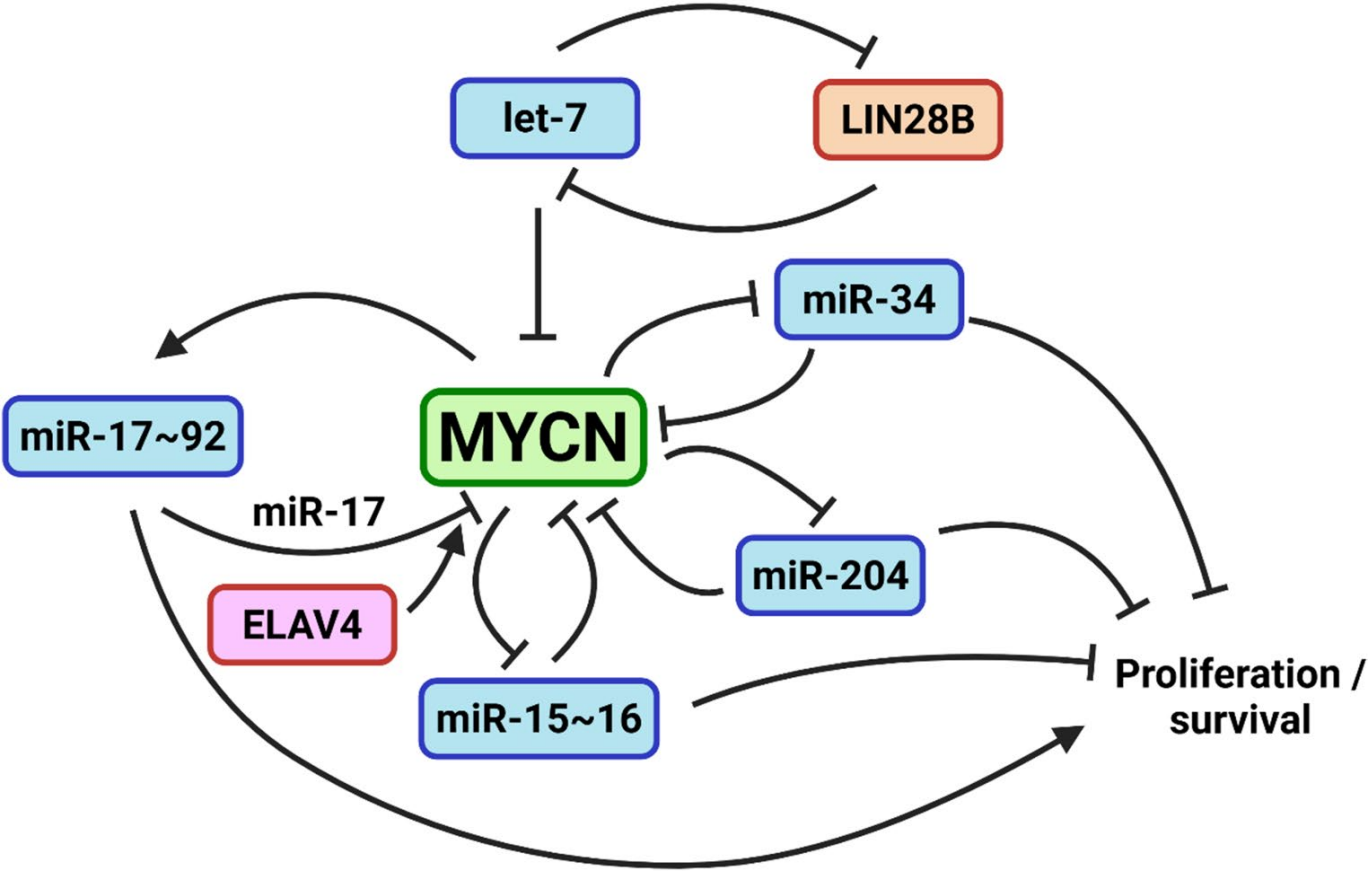
MYCN is a hub for microRNA: transcription factor co-regulation. The MYCN 3’UTR possesses binding sites for almost 200 miRNAs, of which dozens have been physically shown to bind the MYCN transcript. Examples of the best studied feedback interactions between MYCN and miRNAs are shown. Through various feedback and both coherent and incoherent feedback loops, MYCN promotes pro-oncogenic outcomes which are either supported by (miR-17 ~ 92) or opposed by (let-7, miR-15 ~ 16, miR-204, miR-34) different miRNAs

The regulatory dynamics surrounding the MYCN gene are complex, involving a broad network of miRNAs that both regulate and are regulated by MYC (Fig. 4). For example, a systematic screen of 470 miRNAs found 29 direct regulators of the MYCN 3’UTR, including 12 whose expression is inversely correlated with that of MYCN across NB tumours [125]. Another study catalogued MYCN 3’UTR-binding miRNAs, identifying dozens in NB and glioblastoma cells, of which members of the miR-17 ~ 92 cluster were the most enriched [106].

These relationships are particularly evident in MYCN-amplified NBs, where altered miRNA signatures are common and tend to become more pronounced with disease progression and the accrual of genomic rearrangements. In the case of miR-34a, direct targeting of MYCN not only impacts its role in cell cycle regulation and thus, tumour aggressiveness [88, 89, 126] but also triggers a cascade of regulatory effects. For example, the suppression of MYCN in turn downregulates genes driving cell proliferation and survival, such as CCND1 and CDK4, leading to inhibited growth and increased apoptosis in NB cells [126]. The aforementioned polycistronic miR-17 ~ 92 cluster is also transcriptionally activated by MYCN [71, 76] which, at least in part, promotes NB tumourigenesis through miR-17-mediated suppression of p21, a key target of the TGF-β signalling pathway and well established tumour suppressor on account of its role in suppressing cell cycle progression [76]. More broadly, miR-17 ~ 92 perturbation resulted in transcriptomic signatures associated with TGF-β, RAS and estrogen signalling [127]. Accordingly, estrogen receptor α (ESR1) was one of several steroid receptors targeted by the miR-17 ~ 92 family, offering an explanation for MYCN amplification diminishing NB responsiveness to estrogen, a known inducer of differentiation. Similarly, suppression of MYCN in NB models leads to the upregulation of the glucocorticoid receptor (GR), which inhibits tumour progression and promotes differentiation [128]. In keeping with the theme of reciprocal feedback, miR-17 itself has been reported to directly target MYCN, though even this is further modulated by competitive binding to the MYCN 3’UTR between miR-17 and ELAVL4 (HuD), an RNA binding protein that regulates MYCN processing and promotes transcript stability [129].

The above example of miR-17 ~ 92 is illustrative of miRNAs whose transcription is directly activated by MYCN and which propagate its oncogenic effects. Alternately, tumour suppressive miRNAs are also associated with MYCN in mutually repressive feedback loops. For example, N-MYC represses the miR-335 gene promoter and thus de-represses genes that would otherwise be targeted by miR-335. These include ROCK1, MAPK1 and LRG1 which are all associated with non-canonical TGF-β signalling, and which promote phosphorylation of the motor protein myosin light chain (MLC), resulting in actin polymerisation, the promotion of myosin ATPase activity and ultimately a contractile force necessary for cell motility [130]. The miR-15/−16 cluster [131] and miR-204 [113] are similarly targeted by, and themselves directly target, MYCN. In both cases, forced miRNA expression opposes pro-tumourigenic effects of MYCN and slows NB growth both in vitro and in vivo.

### LIN28B - miRNA - MYCN interactions

One especially important and complex feedforward interaction involving MYCN is illustrated by a GRN also involving the LIN28B RNA binding protein (RBP) and the let-7 family of miRNAs. The LIN28 family is widely implicated in the maintenance of “stemness” [132] and together with key factors like the Yamanaka TFs NANOG, OCT4, and SOX2, can reprogram differentiated cells back to a pluripotent state [133]. LIN28B is frequently overexpressed in high-risk NB and is intimately connected to MYCN and miRNA pathways by virtue of its role in miRNA maturation. LIN28B binds to the terminal loop of pri- and pre-let-7 and blocks let-7 maturation [134], in so doing de-repressing let-7 targets. This includes a complex regulatory network involving the GTPase RAN, RAN-binding protein-2 (RANBP2) and the kinase AURKA whose activity is RAN-dependent. Through this network, high LIN28B increases RAN expression and represses let-7 which in turn de-repress RANBP2, AURKA and MYCN. AURKA, itself a potential therapeutic target in NB, is then free to phosphorylate and activate MYCN, as well as other pro-migratory downstream targets including ROCK1 and MAPK1 [135]. In turn, MYCN itself drives LIN28B expression, thus forming a feedback loop that exacerbates the disease by further suppressing let-7 miRNAs and sustaining high levels of MYCN, promoting the proliferation and maintenance of tumour cells [136]. To add even further complexity, MYCN mRNA has been reported to act as a ceRNA for let-7, as its amplification can achieve such a level as to represent almost 20% of all let-7 targeted transcripts in some MYCN-overexpressing NB. By binding to let-7, this excess of MYCN mRNA occupies the miRNA and thus de-represses other let-7 targets which further reduces let-7’s tumour suppressive activity and promotes oncogenesis [136, 137].

### PHOX2B - miRNA interaction

Of the many targets of MYCN, one prominent example in the context of sympathetic neuronal development is Paired – like homeobox 2B (PHOX2B), a TF that is essential for the early development of the autonomic nervous system. PHOX2B is highly expressed in the sympathetic, parasympathetic, and enteric ganglia of the developing autonomic nervous system, where it plays a critical role in the regulation of pathways such as Delta–Notch signalling to promote neuronal differentiation [138]. Although rare, mutations in PHOX2B can result in missense or frameshift alterations that disrupt normal protein function and lead to aberrant activation of Delta–Notch signalling and impaired neuronal differentiation [139]. More commonly, however, dysregulation is driven by PHOX2B over-expression [140]. Again, miRNAs play important regulatory roles. miR-204 is particularly pivotal, where it acts as a tumour suppressor in NB by directly targeting both PHOX2B [141] and MYCN [113]. At least in the case of MYCN, reciprocal regulation is evident with the miR-204 promoter also suppressed by MYCN binding [113]. In NB, miR-204 is frequently under expressed, leading to the unchecked activity of PHOX2B and MYCN. Correspondingly, miR-204 over-expression inhibits NB cell proliferation in vitro and tumourigenesis in vivo [113]. PHOX2B is also reported to be directly suppressed by additional tumour suppressive miRNAs. Both miR-99b-5p [104] and miR-125a [142] have been shown to target PHOX2B directly through binding to its 3′ UTR and the over-expression of both miRNAs reduces NB proliferation and viability, both in cell lines and xenograft models. In the case of miR-99b-5p, the introduction of miRNA mimics also sensitises NB cells to doxorubicin, and indirectly reduces LIN28B expression, suggesting a broader impact on MYCN-related oncogenic pathways.

### Other TF - miRNA interactions

Although key TFs such as MYCN and PHOX2B have been a focus of many investigations, they are by no means the only examples of TFs under miRNA-mediated control that are relevant to developmental processes and NB. For example, The TF ELF4 maintains NB cells in a proliferative, undifferentiated state through its direct regulation of cell cycle genes, an effect reversed by miR-124 with which ELF4 participates in a dual negative feedback loop [95]. miR-124 also inhibits cell cycle progression in NB via the silencing of a battery of cytoskeletal transcripts [143] and is capable of inducing NB cell differentiation by directly targeting the TF AHR (Aryl Hydrocarbon Receptor) [144]. Other examples of tumour suppressor roles attributed to the targeting of TFs by miRNAs in NB include miR-9 and miR-103 restraining ID2 expression [145], miR-205 targeting CREB1 [146] and miR-27b targeting PPARγ [147]. Such studies highlight that in addition to the key TFs such as MYCN and PHOX2B, there are likely a number of miRNA-TF regulatory axes that operate in NB or similar developmental processes that are yet to be uncovered, and their potential for therapeutic intervention yet to be fully explored. It also raises an interesting question: Does the vast extent of reports describing interactions between miRNAs and MYCN indicate that MYCN is a genuine hub of miRNA-mediated regulation, or does the number of reports simply reflect a high volume of research driven by the known importance of the gene? As the field advances, extensive miRNA regulation might similarly be characterized for other TFs and open new avenues for therapeutic targeting.

### Regulation of the adrenergic-mesenchymal switch in neuroblastoma

One of the factors that may be driving the existence of a multitude of regulators is the distinct states that tumours can adopt. In particular, NB cells, and other peripheral neuroblastic tumours with neural crest origins (ganglionueuroblastoma, ganglioneuroma), are known to switch between adrenergic (ADRN) and mesenchymal (MES – NCC-like) phenotypes; two distinct cellular states characterized by unique transcriptional profiles and functions [17, 31, 148]. These states build upon earlier morphological classifications of NB cells into “N” and “S” types: “N” cells, which are small and rounded with neurite-like processes, align with the ADRN phenotype, while “S” cells, which are larger and more substrate-adherent, resemble MES cells [29, 149]. This ADRN-MES transition involves extensive gene regulatory programs that result in cells with vastly different capacities for proliferation, differentiation and response to therapy. MES cells are associated with a drug-resistant phenotype, a gene expression profile consistent with a less differentiated cell population and an ability to promote tumour aggressiveness and relapse [9, 31, 34, 35, 150, 151]. Accordingly, therapeutic interventions like chemotherapy enrich for treatment resistant MES cells. This suggests that regulatory reprogramming allows NB cells to adapt to environmental pressures and presents further challenges for therapeutic intervention.

As with EMT, the interplay between the adrenergic and MES modules is not binary but reflects a continuum, with individual cells expressing elements of both modalities, further contributing to NB heterogeneity and regulatory complexity [148, 152]. Using both bulk and single-cell profiling, it has been observed that even when tumours or xenografts are initiated from cells enriched for one identity (e.g., MES), they often shift toward a predominantly ADRN phenotype in vivo, indicating a strong influence from microenvironmental cues [32]. Nevertheless, MES-like cells persist as a minority in patient samples, suggesting ongoing plasticity and potential reservoirs of therapy-resistant phenotypes [153].

This transition between ADRN and MES states is tightly regulated by networks of TFs and miRNAs, which must work in coordination to maintain cellular identity or facilitate switching driven by environmental or therapeutic pressures. Each cellular state, ADRN or MES, demands specific transcriptional programs that are executed through a combination of master TFs and miRNA-mediated fine-tuning. For instance, of the hundreds of differentially expressed genes between ADRN and MES cell states, 18 and 20 respectively were TFs. ADRN-associated TFs like PHOX2B, HAND2,GATA3 and SOX11 are pivotal in maintaining the adrenergic phenotype, while MES cells rely on TFs such as AP-1 family members (e.g., FOS, JUN) to drive their transcriptional identity [17, 154].

In terms of the ADRN-MES network, miR-124 and miR-335 are the most highly implicated miRNAs. miR-124, a neurogenic miRNA enriched in ADRN-like cells, drives neuronal differentiation and stabilizes the ADRN phenotype [155]. MiR-124-3p directly targets mesenchymal and cytoskeletal genes such as MYH9, ACTN4, and PLEC, reversing resistant phenotypes, reducing invasiveness and enhancing chemotherapy sensitivity [143]. Conversely, miR-335 is enriched in MES cells and plays a pivotal role in maintaining their identity by suppressing HAND1, a critical regulator of neural crest differentiation, and modulating Notch signalling through JAG1, a cell-surface ligand that activates Notch receptors [156]. This reciprocal regulation of miR-124 and miR-335 is reminiscent of EMT-associated GRNs (including miR-200 and ZEB) and highlights both the dynamic interplay between miRNAs and transcriptional programs, and the frequency with which such networks facilitate state transitions.

Despite increasing recognition of ADRN–MES plasticity, the definitive identification of bona fide malignant MES-like cells in patient NB tumours remains unresolved. For instance, most evidence for MES programs arises from in vitro models or post-treatment contexts, and emerging single-cell studies suggest that MES-like transcriptional states may often reflect stromal cells rather than tumour cells. In some cases, weak or hybrid MES signatures have been detected in ADRN cells under therapeutic pressure, but these appear transient and rare. Thus, distinguishing true malignant MES cells from non-malignant mesenchymal populations remains a critical challenge, likely requiring spatial or lineage-based validation [36].

### Therapeutic potential of MicroRNAs in neuroblastoma

Despite the availability of multimodal treatments for NB, including high-dose chemotherapy, surgery, radiation and immunotherapy, the therapeutic regimens, particularly chemo and radiation therapy, remain highly toxic and are associated with severe long-term side effects, such as developmental delays, organ dysfunction, and an increased risk of secondary malignancies [157]. To match patients with an appropriate level of therapeutic intervention, patients are stratified into different risk groups based upon such factors as age, disease stage at diagnosis, genetic aberrations (e.g., MYCN amplification) and histological features. For instance, low-risk NB patients may be managed with surgery alone or minimal chemotherapy [157] while intermediate-risk patients receive the COJEC regimen (carboplatin, etoposide, cyclophosphamide, vincristine) followed by surgery. However, high-risk NB patients undergo aggressive treatment protocols that include induction chemotherapy to shrink tumours, consolidation with high-dose chemotherapy and autologous stem cell transplantation (ASCT), immunotherapy using anti-GD2 monoclonal antibodies (targeted against the GD2 sphingolipid present on NB cell surfaces) and differentiation agents like retinoic acid (RA) [124]. Additionally, targeted therapies such as ALK inhibitors are administered to patients harbouring ALK mutations or amplifications. These constitute less than 10% of NB cases at diagnosis, but are more prevalent at relapse [158]. While ALK inhibitors have demonstrated efficacy in ALK-mutant NB subsets, their effectiveness is confined to these specific genetic backgrounds, underscoring the importance of developing further precision medicine approaches. Despite these advancements, approximately half of high-risk patients either do not respond adequately to first-line therapies or relapse within two years post-treatment, highlighting the limitations of current treatment modalities and the pressing need for novel, less toxic, and more effective therapeutic strategies.

Immunotherapy offers one such avenue for treatment with potentially reduced genotoxicity, though it faces its own set of challenges specific to NB. For example, due to the tumour’s low mutational burden, traditional immune checkpoint inhibitors effective in adult cancers with high mutational loads are less applicable. This limitation necessitates the development of alternative immunotherapeutic strategies, such as targeting overexpressed antigens like GD2, which are minimally expressed in normal tissues but abundant on NB cells. These approaches however must contend with the immunosuppressive tumour microenvironment typical of NB, which is rich in elements like M2-polarized macrophages and myeloid-derived suppressor cells that can significantly inhibit the effectiveness of immune-based therapies [159]. Indeed, in clinical trial GD2-CAR-T cell therapy for children with relapsed or refractory NB failed to meet its clinical objectives [160].

A functional screen in which > 1,200 miRNAs were transfected into NB cells identified miR-99b-5p, miR-380-3p and miR-485-3p as promoting chemosensitivity [104], whilst a CRISPR-Cas9 knockout screen identified miR-1304-5p as a desensitizing agent to ALK-directed tyrosine kinase inhibitors [115]. Given such reports, and advances in chemical modifications and packaging technologies such as Lipid Nanoparticles (LNPs) that enhance small RNA stability and uptake [161], there is hope that miRNAs might be adapted into new therapies. For example, miR-204 which directly suppresses both MYCN and PHOX2B, inhibited viability, migration and spheroid growth when introduced into NB cells [162]. However, In vivo the challenge of tissue-specific delivery remains. At present, the liver is the only organ to which RNA therapies can be reliably and efficiently delivered and is the only organ to which the targeting of siRNA-based therapies have been clinically approved [163]. However, by altering the composition of LNPs, biodistribution and tissue specificity can be altered [164]. In this way, a novel LNP formulation packaging siRNAs against MYCN showed significant retardation of NB xenograft tumour growth and enhanced survival [165]. To increase targeting specificity further, “surface functionalisation” may be employed whereby the nanoparticles outer surface is modified with antibodies, peptides or sugars that bind to cell surface receptors on target cells, promoting receptor-mediated uptake. Pre-clinical success in NB has been reported with siRNAs targeted against PLK1 delivered to NB cells in EGFR-targeting nanoparticles [166]. GD2-targeted nanoparticles have also been used to co-deliver an anti-MYCN siRNA together with doxorubicin [167], or to deliver a combination of both miR-34a and let-7 to NB xenografts [168]. In both cases, in vivo NB suppression was enhanced by the combinatorial nature of the delivered therapies. An alternative approach may also lie with RNA-adjacent strategies whereby small molecules are delivered with the aim of altering NB RNA-associated processes. Whilst in the early stages of development, a screen identified epigallocatechin 3-gallate (EGCG) as reducing NB tumourigenicity by disrupting the LIN28B/let-7 regulatory loop to increase let-7 production and thus, the anti-tumour properties it promotes [169]. Similarly, the kinase inhibitor PP121, itself a robust inducer of miR-124, arrested proliferation and promoted the differentiation of multiple NB cell lines [155].

RNA-based therapies (including miRNAs) offer tremendous potential in that they provide an opportunity to target any gene desired, using a technology which is rapid in terms of both design and synthesis. Whilst challenges remain, not least of which NB-specific targeting, the successes of pre-clinical studies described here offer hope for the expansion of this approach. Similarly, using miRNAs as biomarkers has also been proposed as the stability and availability of miRNAs in body fluids could provide a non-invasive method for real-time monitoring of disease progression, treatment response, and the early detection of relapse [170]. At this stage, no miRNAs have yet achieved the necessary validation and standardization required for clinical use and no RNA-based therapies have been approved for the treatment of NB, however continuing advances provide hope this may change.

## Data Availability

Not applicable.
